# Parvovirus B19 Infection in Pregnancy: Awareness of the Increased Incidence of Severe Intrauterine Infection

**DOI:** 10.3390/diagnostics15111397

**Published:** 2025-05-31

**Authors:** Eleonora Torcia, Alessandra Familiari, Elvira Passananti, Maria Vittoria Alesi, Giulia di Marco, Federica Romanzi, Marco De Santis, Tullio Ghi, Elisa Bevilacqua

**Affiliations:** 1 Department of Women and Child Health Sciences and Public Health, Women Health Area, Fondazione Policlinico Universitario Agostino Gemelli IRCCS, Largo Agostino Gemelli 8, 00168 Rome, Italy; eleonora.torcia1@guest.policlinicogemelli.it (E.T.); alessandra.familiari@policlinicogemelli.it (A.F.); elvira.passananti@gmail.com (E.P.); giulia.dimarco1102@gmail.com (G.d.M.); federica.romanzi@gmail.com (F.R.); marco.desantis@policlinicogemelli.it (M.D.S.); tullio.ghi@policlinicogemelli.it (T.G.); 2 Unit of Obstetrics and Gynecology, Università Cattolica del Sacro Cuore, Largo Agostino Gemelli 8, 00168 Rome, Italy; mariavittoria.alesi@gmail.com

**Keywords:** parvovirus B19, non-immune hydrops fetalis (NIHF), intrauterine erythrocyte transfusion (IUT), infectious disease during pregnancy

## Abstract

In 2024, Europe experienced a significant upsurge in cases of Parvovirus B19 (B19V), the etiological agent of erythema infectiosum, also known as fifth disease. The prevalence of B19V in pregnant women, a particularly vulnerable population, holds critical clinical significance. Typically, B19V follows a well-documented seasonal pattern, with annual epidemics peaking in the spring and larger outbreaks occurring approximately every four years. B19V exhibits a tropism for erythroid precursor cells, potentially resulting in fetal anemia and, in the most severe scenarios, intrauterine demise. Severe in utero infections necessitate intrauterine erythrocyte transfusion (IUT), a highly specialized and technically demanding procedure that is exclusively performed in tertiary-level prenatal care units. This study delineates how the notable increase in B19V infections is also reflected in our prenatal diagnosis unit at Fondazione Policlinico Agostino Gemelli (FPG) IRCCS, Rome, Italy. According to our case series, since 2018, B19V has been identified as the second most common cause of fetal anemia during the study period (29%, 6 patients), yet it accounted for the majority of IUT procedures performed in 2024 (16 out of 19 cases, 84.2%). Given the rising incidence of severe intrauterine infections in recent epidemic cycles, healthcare professionals should maintain a high index of suspicion regarding the clinical manifestations of maternal B19V infection and its potential obstetric complications. Further research is imperative to evaluate the cost-effectiveness of routine screening for B19V immunity in pregnant women and to investigate the long-term neurodevelopmental and clinical outcomes of neonates affected by intrauterine B19V infection.

## 1. Introduction

Parvovirus B19 (B19V) is a small, single-stranded, non-enveloped DNA virus, recognized as the etiological agent of erythema infectiosum, also known as fifth disease [1,2]. B19V infection predominantly manifests in school-aged children, presenting with a characteristic facial rash termed “erythema infectiosum”, accompanied by mild pyrexia, arthralgia, and cephalalgia [3]; however, it frequently remains asymptomatic, particularly in adults [4]. B19V is primarily disseminated via respiratory droplets; nonetheless, hematogenous transmission through blood and blood-derived products has been documented [3]. The incubation period ranges from 4 to 14 days post exposure, although it may extend up to three weeks in certain cases [5]. In non-immune pregnant women acquiring the infection, the probability of vertical transmission to the fetus ranges between 33% and 51% [6]. B19V exhibits a tropism for erythroid precursor cells, myocardial tissue, and placental endothelium [7]. The infection and subsequent lysis of erythroid progenitor cells makes B19V a potent suppressor of hematopoiesis, culminating in transient aplastic crisis, particularly in the context of underlying hemolysis [8,9]. In immunocompetent adults, aplastic crisis is generally well tolerated, with minimal anemia as the immune system determines promptly the clearance of viral infection. However, the fetus exhibits an increased demand for erythrocytes and a larger erythroid mass, characterized by rapid cellular turnover, explaining why it is highly susceptible to disruptions in erythropoiesis. The fetal liver is the primary hematopoietic organ from 9 to 24 weeks of gestation [5]. The second trimester also sees the most rapid increase in the mass of fetal red blood cells. However, the half-life of fetal red blood cells produced in this period is relatively short, approximately 45–70 days [5]. Therefore, the fetus is extremely vulnerable to any pause in red blood cell production during the second trimester and is more susceptible to the changes caused by parvovirus B19 [10]. This risk is greatly reduced in the third trimester when fetal hematopoiesis migrates to the bone marrow, and red blood cell lifespan increases [5]. Fetal anemia, in conjunction with associated hepatitis, hypoalbuminemia, and myocarditis, may precipitate cardiac insufficiency and subsequent hydrops fetalis [11]. Furthermore, the suppression of platelet precursors and direct cytotoxic effects on megakaryocytes can contribute to fetal thrombocytopenia [12,13]. Severe in utero infections necessitate intervention via intrauterine erythrocyte transfusion (IUT), an invasive procedure conducted exclusively in specialized fetal medicine centers [14]. Typically, a minimum of two IUTs are required to manage severe fetal compromises. The significant incidence of concurrent severe fetal thrombocytopenia may substantially influence procedural outcomes due to an elevated risk of fetal exsanguination [12,15]. Various studies recommend ensuring the availability of compatible platelet concentrates at the time of transfusion, and if fetal platelet levels fall below 50 × 10^9^/L, platelet transfusion should be performed to mitigate the risk of catastrophic hemorrhage [12,15]. Survival rates following IUT for B19V infection range from 67% to 84%, in contrast to 30% to 50% in pregnancies without in utero therapeutic intervention [6]. Prognosis is strongly correlated with the severity of fetal anemia and the presence of hydrops. While IUT enhances survival outcomes, fetuses with advanced hydrops often exhibit suboptimal responses to treatment. In such cases, myocarditis may represent a pivotal determinant of fetal survival [16]. Myocardial dysfunction secondary to viral-induced myocarditis may explain instances where hydrops fails to resolve or exacerbates post IUT. Indeed, blood transfusion may further compromise fetal cardiovascular homeostasis, potentially culminating in fetal demise. Some investigations have identified leukocytosis on cordocentesis blood analysis as a robust biomarker of myocarditis, portending a poor prognosis [17]. Current evidence underscores the necessity of meticulous fetal surveillance in pregnancies complicated by maternal B19V infection. Ultrasound monitoring is imperative for detecting signs of fetal anemia, notably an increased peak systolic velocity of the middle cerebral artery (PSV-MCA) on a Doppler assessment (>1.5 multiples of the median, MoM) and sonographic markers of hydrops (ascites, skin edema, pleural and pericardial effusions, and placentomegaly) [5]. The International Society of Ultrasound in Obstetrics and Gynecology (ISUOG) guidelines for congenital infections advocate serial ultrasonographic assessment in all women testing positive for B19V-specific IgM antibodies, irrespective of IgG status, to ascertain potential fetal involvement [18]. Notably, serological testing exhibits a considerable false-negative rate (20–40%), particularly in early asymptomatic stages when high viral loads may form immune complexes with B19V-specific antibodies, rendering them undetectable [19]. Therefore, in cases of strong clinical suspicion, despite negative IgM results, molecular diagnostic techniques such as polymerase chain reaction (PCR), IgG avidity testing, or amniocentesis for viral DNA detection should be employed [3,18,19]. Once maternal infection is confirmed, weekly PSV-MCA evaluations should be performed, with IUT indicated when PSV-MCA exceeds 1.5 MoM and/or when other sonographic markers of severe fetal anemia (e.g., hydrops) are identified [18]. Continuous fetal surveillance is warranted for a minimum of 8 to 12 weeks post maternal infection [13,14,20,21]. If, after 12 weeks from exposure, no abnormal ultrasound findings suggestive of B19V-related complications are observed, the probability of adverse fetal outcomes is significantly diminished [18,22,23].

Epidemiologically, B19V infections follow a seasonal trend, with annual epidemics peaking in spring and major outbreaks occurring approximately every four years [14]. A clear association exists between epidemic years and an increased incidence of severe intrauterine infections [6,14]. The risk of intrauterine fetal demise (IUFD) is estimated at 12 per 100,000 pregnancies in non-epidemic periods, surging to 48 per 100,000 during epidemic years [14]. Recent investigations have highlighted the substantial impact of COVID-19-related restrictions on the incidence and seasonal variability of airborne-transmitted viruses, including B19V [24,25]. The marked decline in B19V circulation during the COVID-19 pandemic has likely contributed to diminished population immunity, potentially predisposing individuals to the resurgence of outbreaks in subsequent years [26].

## 2. The Third-Level Birth Center’s Experience

In 2024, a pronounced upsurge in B19V infections was documented across Europe and the United States, prompting national centers for disease prevention to advise clinicians to maintain a heightened level of clinical vigilance for pregnant women exhibiting suggestive symptomatology or recent exposure to confirmed cases [27]. Similarly, at the Fetal Medicine Unit of Fondazione Policlinico Agostino Gemelli IRCCS in Rome, Italy, we observed a substantial escalation in severe intrauterine B19V infections, with an increased incidence of adverse perinatal outcomes surpassing prior expectations based on historical data. We conducted a retrospective cohort analysis of all cases undergoing IUT between 2018 and 2024. Throughout the study period, a total of 21 patients received IUT, with 58 invasive procedures performed in total. These interventions were primarily executed by three different senior fetal medicine specialists. The predominant etiology of severe fetal anemia requiring IUT was anti-D alloimmunization, accounting for 57% of patients (12 out of 21). The second most prevalent cause was B19V infection, responsible of severe fetal anemia in 29% of patients (6 out of 21) who received a IUT and they were all recorded in 2024. In 2024, B19V infection emerged as the leading cause of severe fetal anemia necessitating IUT, accounting for 16 out of 19 procedures (84.2%), as illustrated in Table 1. Notably, the highest frequency of IUT procedures was recorded in 2024, with 19 procedures performed, constituting 32.7% of all IUTs conducted throughout the study period. Among these six patients who required a IUT for B19V, in four (Cases 1, 3, 4 and 6) out of the six patients, serological testing for Parvovirus B19 (B19V) was prompted by the immediate presentation of severe signs of fetal anemia, as hydrops fetalis and elevated PSV in the MCA, at the ultrasound. These clinical findings raised suspicion of intrauterine infection and led to a targeted serological investigation. The remaining two patients (Cases 2 and 5) were referred to our fetal medicine unit following confirmed exposure to B19V, in both cases through close contact with their own symptomatic children. For each patient at the admission to the Fetal Medicine Unite, we performed serology IgG and IgM and PCR for B19V, which all produced positive results. For the detection of Parvovirus B19 antibodies, our laboratory employed the LIAISON^®^ Biotrin Parvovirus B19 IgG and IgM Plus panel, a chemiluminescent immunoassay (CLIA) designed for the quantitative determination of specific IgG antibodies (expressed in IU/mL) and the qualitative assessment of IgM antibodies (expressed as S/CO) in human serum. The detection of parvovirus B19 DNA was performed using the QIAGEN artus^®^ Parvo B19 PCR kit. As mentioned, B19V-specific IgG antibodies are detected using a quantitative assay, with values exceeding 2.5 IU/mL considered indicative of a positive result, while IgM antibodies are assessed qualitatively, with positivity defined by a signal-to-cut-off (S/CO) ratio greater than 1.10. Parvovirus B19 DNA is detected through a quantitative PCR assay, with a positivity threshold set at >140 IU/mL. The key clinical parameters, sonographic findings, laboratory results, and perinatal outcomes of pregnancies complicated by B19V infection in 2024 are summarized in Table 2. Based on our institutional experience, the overall survival rate following IUT was 50%, with three cases of intrauterine fetal demise (IUFD) and three liveborn neonates. The high rate of adverse outcomes can be related to the severity of case presentation. As detailed in Table 2, among neonates delivered following B19V infection and treated with IUT, the most adverse neonatal outcome was documented in Case 4, who exhibited fetal growth restriction (FGR) and neonatal respiratory distress syndrome (RDS) at birth. In this patient, B19V infection occurred at an earlier gestational age (18 + 6 weeks), with profound fetal anemia (PSV-MCA 2 MoM) and hydrops fetalis, necessitating a total of five IUTs—the highest number of IUTs performed in a single pregnancy within our case series.

## 3. Recommendations

**Figure 1:**
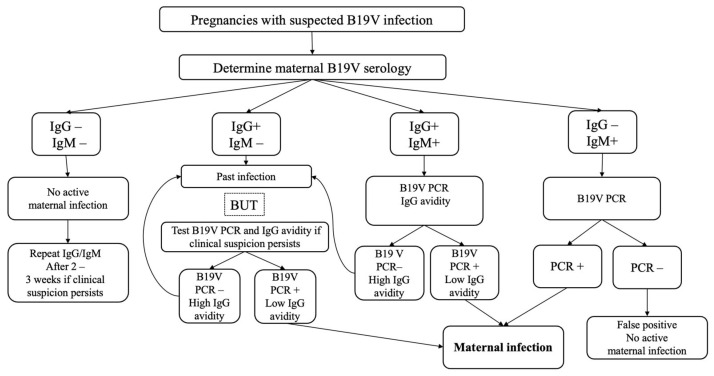
Flow diagram for diagnosis in pregnancies with suspected B19V infection.

In conclusion, clinicians should maintain a high degree of vigilance regarding the clinical presentation and associated risks of B19V infection during pregnancy, particularly in epidemic years. Serological screening for B19V should be considered in pregnant women presenting with febrile illness, exanthema, or debilitating arthralgia; those with documented exposure to individuals infected with B19V; and in any instance where ultrasonographic evaluation reveals fetal hydrops [6]. When Parvovirus B19 infection is confirmed, patients should be promptly referred to a specialized fetal medicine center for appropriate follow-up and timely intervention in the event that severe signs of fetal anemia are detected. Current evidence regarding the long-term prognosis of infected fetuses remains limited; however, according to the latest studies, the risk of abnormal neurodevelopment appears to be low (approximately 10%) in hydropic fetuses and negligible in non-hydropic cases [10,28]. Figure 1 represents a flowchart outlining the proposed strategy for the diagnosis in pregnancies with suspected B19V infection. Further research is warranted to assess the cost-effectiveness of universal screening for B19V immunity in pregnant women, as well as to elucidate the long-term neurodevelopmental and clinical outcomes of neonates affected by intrauterine B19V infection.

## Figures and Tables

**Table 1:** IUTs performed at FPG per year from 2018 to 2024 and their cause.

Year	n° IUT	Causes
2018	12	10 anti-D alloimmunization 2 idiopathic
2019	0	
2020	15	13 anti-D alloimmunization 2 idiopathic
2021	5	3 anti-Kell alloimmunization 2 anti-D alloimmunization
2022	7	5 idiopathic 2 anti-D alloimmunisation
2023	0	
2024	19	16 ParvovirusB19 2 anti D alloimmunisation 1 idiopathic

**Table 2:** Main characteristics of pregnancies complicated by Parvovirus B19 infection that underwent IUT in 2024, such as gestational age (GA) at diagnosis, serology (IgM and IgG) and PCR for B19 at the diagnosis, ultrasonographic features like peak systolic velocity of the middle cerebral artery, the presence (+) or absence (−) of hydrops, gestational age (GA) at the first IUT, how many IUTs were performed for each patients, the localization of the placenta if it was anterior (A) or posterior (P) and the obstetrical outcome. In case of intrauterine fetal death (IUFD) (+), we reported the gestational age (GA) of demise.

Patient n°	GA Diagnosis	IgM B19V (S/CO)	IgG B19V (UI/mL	PCR B19V (UI/mL)	PSV MCA	MoM	Hydrops	GA IUT	N° IUT TOT.	Placenta	IUFD	GA Demise	Neonatal Outcome
Case 1	25 + 0	2.6	27	75,600	73	2.4	+	25	3	A			Born full term, good clinical conditions.
Case 2	21 + 2	7.8	65.5	7,000,000	41	1.52	−	21 + 2	1	A			Born full term, good clinical conditions.
Case 3	19 + 2	21.8	101	62,940,500	42	1.68	+	19 + 5	1	P	+	19 + 6	
Case 4	18 + 6	5.86	48.20	9,215,500	56	2	+	19 + 0	5	P			Born at term, with FGR and RDS at birth.
Case 5	19 + 0	2.66	29.1	/	48	1.8	−	21 + 1	3	P	+	24 + 3	
Case 6	12	38.1	4.5	>70,000,000		2.02	+	21 + 2	3	A	+	23 + 1	

## Data Availability

The raw data supporting the conclusions of this article will be made available by the authors on request.
